# The Anterior Cingulate Gyrus and Social Cognition: Tracking the Motivation of Others

**DOI:** 10.1016/j.neuron.2016.04.018

**Published:** 2016-05-18

**Authors:** Matthew A.J. Apps, Matthew F.S. Rushworth, Steve W.C. Chang

**Affiliations:** 1 Department of Experimental Psychology, University of Oxford, Oxford, OX1 3UD, UK; 2 Nuffield Department of Clinical Neuroscience, University of Oxford, Oxford, OX3 9DU, UK; 3 Department of Psychology, Yale University, New Haven, CT 06520-8205, USA; 4 Department of Neuroscience, Yale University School of Medicine, New Haven, CT 06520-8001, USA

## Abstract

The anterior cingulate cortex (ACC) is implicated in a broad range of behaviors and cognitive processes, but it has been unclear what contribution, if any, the ACC makes to social behavior. We argue that anatomical and functional evidence suggests that a specific sub-region of ACC—in the gyrus (ACCg)—plays a crucial role in processing social information. We propose that the computational properties of the ACCg support a contribution to social cognition by estimating how motivated other individuals are and dynamically updating those estimates when further evidence suggests they have been erroneous. Notably this model, based on vicarious motivation and error processing, provides a unified account of neurophysiological and neuroimaging evidence that the ACCg is sensitive to costs, benefits, and errors during social interactions. Furthermore, it makes specific, testable predictions about a key mechanism that may underpin variability in socio-cognitive abilities in health and disease.

## Highlights

• The anterior cingulate gyrus (ACCg) mainly signals other-oriented information
• The ACCg responds to others’ motivation and prediction errors
• ACCg variability may lead to variability in social cognition in health and disease
• A computational framework is proposed accounting for ACCg social responses

## Teaser

Apps et al. review specialized properties of the anterior cingulate gyrus in social cognition and put forward a computational account of how the motivation of other agents is estimated in the anterior cingulate gyrus of human and non-human primates.

## Main Text

### Introduction

Accounts of the anterior cingulate cortex (ACC) have highlighted its role in fundamental cognitive processes, including motivation, decision making, learning, cost-benefit calculation, as well as conflict and error monitoring (Holroyd and McClure, 2015, Holroyd and Yeung, 2012, Rushworth et al., 2012, Kolling et al., 2016, Laubach et al., 2015, Shackman et al., 2011, Shenhav et al., 2013, Ullsperger et al., 2014, Verguts et al., 2015). While such theories have dominated research on ACC, there is also a long, if less influential, tradition associating ACC with social behavior. For example, many years ago, links were drawn between ACC and the basic affiliative and communicative behaviors in which all mammals engage (MacLean, 1993). That link has persisted even as the neuroscientific approaches and behavioral paradigms used to investigate social interaction have increased dramatically in their sophistication (Saxe, 2006, Schilbach et al., 2013, Somerville et al., 2006, Wang et al., 2011, Haroush and Williams, 2015). It is apparent when neuroimaging is used to examine human brain activity when people engage in socially oriented modes of cognition, such as empathy (Singer et al., 2004, Lamm et al., 2011), that might once have been thought recalcitrant to neuroscientific examination. In addition, disorders of social cognition have long been linked to the structure and function of the ACC (Apps et al., 2013b, Anderson and Kiehl, 2012). Understanding the mechanistic contribution of this region to social behavior is therefore vital for understanding social cognition in health and disease.

It is sometimes assumed that any role ACC has in processing social information simply reflects an aspect of more generalized cognitive control processes. Here we review recent experiments, primarily in primates such as macaques and humans, to examine whether this is indeed the case. Drawing on anatomical, lesion, single-unit recording, and neuroimaging studies, we suggest that the notion that social information processing in the ACC simply reflects general cognitive control processes can be refuted. Anatomical evidence points to considerable heterogeneity in ACC cytoarchitecture and connectivity. Such heterogeneity is indicative of functions being localized to discrete sub-regions. We highlight how evidence points to social information being processed in a specific ACC sub-region in the gyrus (ACCg). Both single-unit recording and neuroimaging studies suggest that this region computes “other-oriented” information (i.e., information about other agents that might be animals or people, rather than ourselves; Apps et al., 2013b, Behrens et al., 2009). We also review this region’s connectional and functional properties in health and in disorders of social cognition before finally describing a model of how this region might compute information that guides our understanding of how motivated other people are. We suggest that this computational framework has potential for providing mechanistic understanding of variability in social behavior in healthy people and also disorders of social cognition.

### Using Social Information to Guide Learning and Decision Making in ACC

Classical as well as recent accounts of the ACC have often posited that this region plays a vital role in processing rewards and also in decision making (Ullsperger et al., 2014, Kolling et al., 2016). Recent work has highlighted that this region may also be particularly important for evaluating cost-benefit information that influences how motivated we are and foraging decisions such as whether to maintain or switch behavior (Holroyd and Yeung, 2012, Kolling et al., 2016). The ACC encodes variables determining the reward rate of a behavior, including its probability of reward and its effortfulness, which are key determinants of how vigorously behaviors should be pursued (Kennerley et al., 2009, Kurzban et al., 2013, Kolling et al., 2016, Rushworth et al., 2012). On first glance it may therefore seem surprising to suggest that this region is also particularly important for processing information about others. However, from an ecological perspective, it isn’t surprising at all. Despite major differences in avian and mammalian neuroanatomy, in birds, species in which foraging is sophisticated, information about the presence of conspecifics is processed in adjacent regions to those concerned with both the costs and benefits of the bird’s own behavior (Aoki et al., 2006, Amita and Matsushima, 2014). Moreover, in humans, social information is processed in the same or adjacent brain structures as those that are engaged during foraging decisions, which may in part be because information about competitors also crucially determines foraging decisions (Mobbs et al., 2013). Such a confluence of information appears to be a general principle of neural circuits for decision making.

Electrophysiological recordings in rodents suggest that neurons in a homologous region to human ACC are concerned with cost-benefit decisions made in social contexts and with learning through observation of others. Hillman and Bilkey (2012) recorded from the ACC as rats evaluated the costs and benefits of competing with a conspecific for a reward. They found that a large proportion of ACC neurons coded the net value (benefit minus cost) of competing for a reward but did not respond similarly to equal levels of reward when no competition was required. Lesions of the same ACC region (Cg1/Cg2) in rats disrupt both cost-benefit decision making (Rudebeck et al., 2006a) and diminish the normal interest they take in other rats (Rudebeck et al., 2007). In mice, the typical “freezing” response when receiving painful stimulation can also be learned by observation. Jeon and colleagues (2010) showed, however, that when the ACC of mice is inactivated, they are no longer able to learn the freezing response through observation. Inactivation of the lateral amygdala did not abolish the ability to learn from observation in the same way. This would suggest that the ACC may have a particularly important role in learning about the behavior of conspecifics in rodents as well cost-benefit information about an animal’s own behavior.

Over the last decade many neuroimaging studies in humans have shown that ACC is active when people interact with others during economic games in which, typically, the experimental participants make decisions that affect not only their own subsequent payoff but also the payoff that other players will receive (Behrens et al., 2008, King-Casas et al., 2005, Gabay et al., 2014, Sanfey et al., 2003, Tomlin et al., 2006, Ruff and Fehr, 2014). A recent study suggested that such responses in the ACC may be driven by neurons that are predictive of the behaviors of another. Haroush and Williams (2015) recorded from the ACC as monkeys performed a similar type of game to those used in human neuroimaging studies—the prisoner’s dilemma game. In this game, players have to choose between cooperating with another or defecting—failing to cooperate. Cooperating resulted in a medium-sized reward for the monkey but only if the other cooperated too; the monkey only received a small reward if it cooperated and the other defected. Defecting led to a large reward payoff although, again, the payoff depended on what the other did too. Not surprisingly ACC neurons responded to the monkey’s own decisions of whether to cooperate or defect but intriguingly they found a second class of neurons predicting the decisions of the other monkey. Such findings suggest some specificity for monitoring and predicting the behavior of others within ACC. However, such experiments give rise to important questions: (1) is such activity a corollary of the social interaction that such tasks entail, or (2) is it simply the case that any difficult decision making, whether it involves other people or not, is associated with ACC activity? After all there are many reports linking ACC to decision making even in the absence of social context, so at the heart of this Review is the question: is there anything special about responses in the ACC when tracking others’ behavior?

If primate ACC activity tracks the behavior of others, then we might expect ACC lesions to disrupt social behavior. It is indeed the case that lesions in humans that impact on large areas of medial prefrontal cortex, including portions of the ACC, result in changes in social behavior, social decision making, and the ability to learn from observation (Blair and Cipolotti, 2000, Krajbich et al., 2009, Anderson et al., 1999, Moretti et al., 2009, Kumaran et al., 2015, Hornak et al., 2003). However, the identity of the key areas that, if damaged, cause social impairments has been difficult to determine because the lesions are large and directly affect several anatomical areas and undercut the connections of even more. In macaques, circumscribed lesions to the ACC, more so than some other frontal areas, disrupt the processing of social information and lead to changes in social behaviors (Hadland et al., 2003, Rudebeck et al., 2006b, Rudebeck et al., 2007, Noonan et al., 2010). There is therefore evidence of a causal link between social behavior and the ACC.

Taken together, research points to the ACC playing a key role not just in reward-guided learning and decision making but also in social cognition. However, to truly understand the “reference frame” in which the ACC responds during social interactions and to examine the specificity of any region for processing social information, it is important to use tasks where such features can be disentangled. In the following sections, we consider some simpler tasks that allow us to examine how ACC activity tracks specific features of others’ behavior and the motivations that inform those behaviors. First, however, we consider the anatomy of the ACC in greater detail and examine whether some of its component zones may have distinct functional properties.

### The Anatomy of the ACC

#### Anatomical Dissociation between the Sulcus and Gyrus

**Figure 1:**
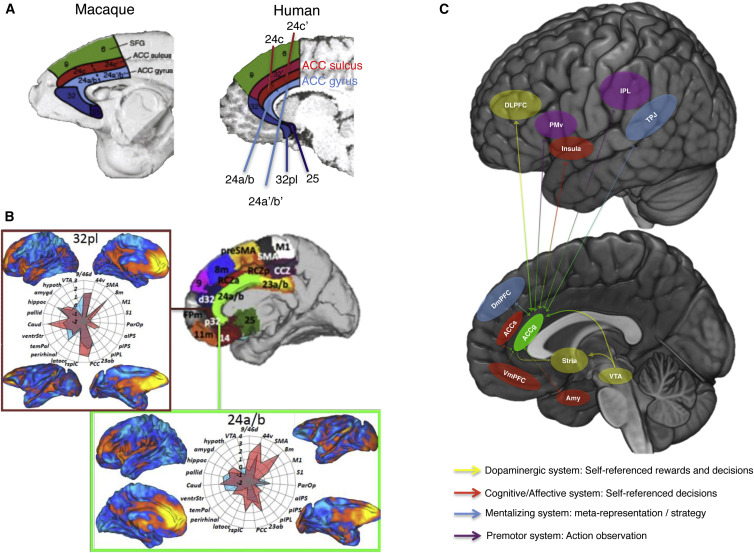
Anatomy of Cingulate Cortex (A) Location of the different regions of the cingulate cortex in the macaque (left) and human (right) brain. Regions in blue comprise the ACC gyrus, red lie in the sulcus (although they may also lie within the adjacent paracingulate gyrus and paracingulate sulcus when they are present). Note that these representative drawings are based on a composite of cytoarchitectonic atlases of the cingulate. The border between areas 24a/b has no gross anatomical landmark and also varies considerably between individuals (Vogt, 2009). Regions in green correspond to those typically referred to as dmPFC. Regions in dark blue are sometimes referred to as part of the vmPFC. Image taken from Rushworth et al. (2004). (B) Connectivity-based parcellation of the medial prefrontal cortex including ACCg (Neubert et al., 2015). The ACC gyrus regions 24a/b and 32pl are shown in light green and maroon, respectively. A distinction between 24a/b and 24a’/b, proposed on the basis of cytoarchitectonic criteria by Vogt (2009) is not always recognized. It was not possible to detect reliable differences between the connectivity profiles of these regions. The green box shows the resting connectivity strength of area 24a/b to other brain areas in both the human (top left: lateral surface; bottom left: medial wall) and macaque (top right: lateral surface; bottom right: medial wall) brain. The central spider plot shows the relative strength of connections between 24a/b and other brain areas in humans (red) and macaques (blue). The maroon box shows the same information for area 32pl. (C) A representative schematic of the connections of the ACCg. The arrows highlight the key systems the ACCg connects to and their putative roles in social cognition. The ACCg (areas 24a/b and 32pl) is connected to a broad set of regions engaged in reward processing, decision making, and social information processing, making it well placed to form part of a distributed network engaged in computing information about the reward-based behavior of others. Abbreviations: VTA, ventral tegmental area; hypoth, hypothalamus; hippoc, hippocampus; amygd/amy, amygdala; pallid, pallidum; Caud, caudate; ventrStr, ventral striatum; temPol, temporal pole; rsplC, retrosplenial cortex; PCC, posterior cingulate cortex; pIPL, posterior inferior parietal lobule; pIPS, posterior intraparietal sulcus; aIPS, anterior intraparietal sulcus; ParOP, parietal operculum; S1, primary somatosensory cortex; M1, primary motor cortex; SMA, supplementary motor area; stria, striatum.

The cingulate cortex in both humans and monkeys contains multiple distinct cytoarchitectonic zones (Palomero-Gallagher et al., 2008, Vogt, 2009) that imply localization of different functions to each zone (Figure 1A). Although many discussions of ACC focus on the anatomical distinctions that arise along the rostro-caudal axis (Silvetti et al., 2014), an equally crucial cytoarchitectonic distinction also exists in the dorsal-ventral dimension between gyral and sulcal regions. Furthermore, differences in connectional anatomy, related to the direction and strength of inter-regional communications, suggest that the ACCg may be functionally distinct from the more frequently studied regions in the adjacent ACC sulcus (ACCs), often referred to as the dorsal ACC (dACC). Notably, the distinction in anatomical properties between the sulcus and gyrus extends across a large extent of the ACC. In many atlases the ACCg contains areas 24a/b and 32 in both humans and macaques, whereas areas 24c and 32′ lie predominantly in the ACCs (Figure 1A).

Throughout this Review, we use the terms ACCg and ACCs to refer broadly to locations anterior to the posterior cingulate cortex (PCC) in the gyrus and sulcus, respectively. We do this because the location of the border between areas 32 and 24a/b has varied across anatomical studies (Walker, 1940, Preuss and Goldman-Rakic, 1991, Carmichael and Price, 1994, Vogt, 2009) and it was not possible to carry out detailed histological analysis of brain tissue in the humans and monkeys in the experiments we discuss. Moreover, although not acknowledged by every authority, there is evidence for cytoarchitectural subdivision within area 24 (24a, a’, b, and b’; Vogt, 2009). However, the anatomical connections of these subdivisions, which constrain and determine function, appear broadly similar throughout 24a/24b (Van Hoesen et al., 1993). In fact many of the connections of both area 32 and 24 are similar (Figure 1B; Van Hoesen et al., 1993, Beckmann et al., 2009, Neubert et al., 2015). Patterns of activity coupling between these areas and the rest of the brain, which only partly reflect monosynaptic connections (O’Reilly et al., 2013), suggest important similarities between human and macaque 24a/b, macaque area 32 and human area 32pl, and the manner in which they interact with the rest of the brain in both species (Neubert et al., 2015; Figure 1B).

Importantly, it has also been argued, on the basis of cytoarchitectural analyses, that there is homology between human and macaque ACC (Preuss and Goldman-Rakic, 1991, Vogt, 2009, Vogt and Paxinos, 2014) and that the ACCs and ACCg differ from one another in similar ways in the two species. This evidence suggests that it may be possible to integrate findings from lesion and neurophysiological studies in monkeys with neuroimaging evidence in humans.

#### The “Social Connectivity” Profile of the ACCg

Humans live in large, complex social groups. It has been argued that living in such large groups has led to evolutionary pressure for larger brains in the primate order. As a result, it is often suggested that there are networks in the brain that have evolved to play important roles in social cognition and behavior (Dunbar and Shultz, 2007, Passingham and Wise, 2012, Chang, 2013, Chang et al., 2013b). Historically, neurobiological models of social cognition have placed importance on three distributed neural networks, each of which may perform different functions that contribute to social behavior (Blair, 2013, Frith and Frith, 2006, Kilner, 2011): (1) a “mentalizing” network involved in inferring others’ mental states and abstract information about social situations, comprising a region in the temporo-parietal junction (TPJ) and the dorsal portions of the medial prefrontal cortex (dmPFC) (Apps and Tsakiris, 2013, Frith and Frith, 2006, Hampton et al., 2008, Lee, 2008, Lee and Seo, 2016); (2) an action observation network (AON) including the ventral premotor cortex (PMv) and adjacent BA44 and the anterior, inferior parietal cortex (Iacoboni and Dapretto, 2006, Kilner, 2011); and (3) a network involved in affective and value-based processing, including the amygdala, ventral portions of the medial prefrontal cortex (vmPFC), and anterior insula (AI) (Blair, 2013, Ruff and Fehr, 2014, Chang et al., 2015, Fareri et al., 2015).

Although there are exceptions, the ACCg has classically not been considered as a part of these networks. Nevertheless, anatomical tracer studies in monkeys and neuroimaging studies in humans examining human brain connections (Figures 1B and 1C) suggest that this region may in fact have strong connections to each of these three social networks (Apps et al., 2013b, Beckmann et al., 2009, Mars et al., 2012, Mesulam and Mufson, 1982, Morecraft et al., 2012, Sallet et al., 2013, Vogt, 2009). This region connects to the TPJ and the dmPFC, which are engaged in mentalizing processes; to several amygdala nuclei, the vmPFC and the AI regions, which are engaged during affective and social processing; and also to the PMv and the inferior parietal cortex regions engaged during action observation. This connectivity profile places the ACCg as a potentially important region that integrates a variety of distinct forms of social information from the different social networks.

Is the connectivity profile of ACCg distinct from other cingulate sub-regions? Notably, both tracer and resting-state studies in macaques and humans highlight that its connectional fingerprint is distinct from that of the adjacent cingulate regions (Beckmann et al., 2009, Margulies et al., 2007). Specifically, none of the connections from the ACCs overlap with those of the ACCg to the mentalizing network or the AON and the connections of the ACCg and the ACCs to the vmPFC and the amygdala also do not fully overlap (Vogt, 2009, Van Hoesen et al., 1993). This would suggest that overall the ACCg has a connectivity profile that plays an important role in processing social information, whereas the ACCs may not have access to all of the same information.

Obviously not all ACCg’s connections are distinct from those of the ACCs. The ACCg also interconnects with many regions to which the ACCs does also. This includes strong interconnections between the ACCg and ACCs, between both regions and several other portions of the prefrontal cortex, as well as parts of the striatum, the parietal lobe, and both the ACCg and ACCs receive monosynaptic input from dopamine neurons in the ventral tegmental area (Vogt, 2009, Williams and Goldman-Rakic, 1998). A diverse array of functions has been ascribed to these regions. However, it is clear that they play important roles in processing the costs and benefits of acting (Floresco et al., 2008, Kolling et al., 2016, Rushworth et al., 2012, Verguts et al., 2015). This would therefore suggest that while the ACCg may be functionally distinct from the ACCs in terms of its role in processing social information, it may also be sensitive to some similar information. This may therefore highlight an important role for the ACCg in processing the costs and benefits of acting in social contexts, much how the ACCs does when evaluating the value of acting for ourselves (Kolling et al., 2016).

### The ACCg and Social Cognition

#### A Functionally Distinct Region of the Cingulate Cortex for Other-Oriented Information?

Lesion studies in macaques suggest that the region of the ACC that is most important for the processing of social information lies in the gyrus. Rudebeck and colleagues (2006b) showed that lesions to the ACCg decreased the value that macaques placed on social stimuli and also led to reductions in the execution of social behaviors. In contrast, lesions to the ACCs, and other parts of the frontal cortex, do not lead to similar changes to the valuation of social stimuli or social behavior, suggesting a special role for ACCg in social cognition (Noonan et al., 2010). Neuroimaging studies in primates provide further support for the notion that the ACCg processes social information. Resting-state connectivity in the ACCg has been shown to correlate with the size of social group in which macaques were living (Sallet et al., 2011, Noonan et al., 2014).

**Figure 2:**
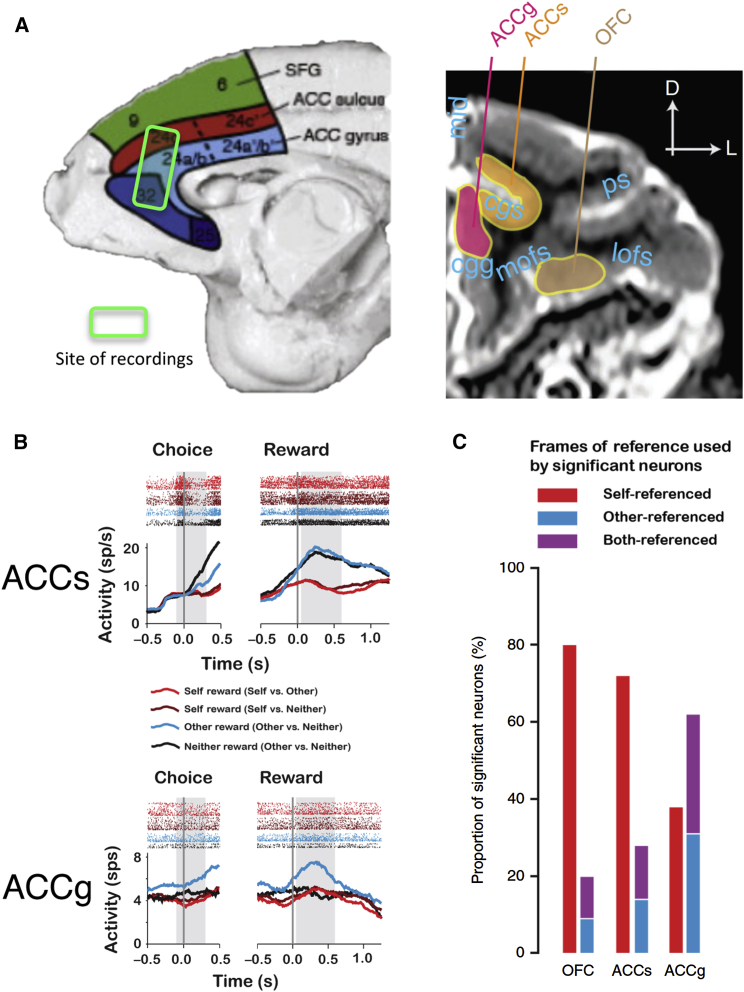
Neurophysiological Recordings from Macaque ACCs, ACCg and OFC as Monkeys Performed a Modified Dictator Game The recipients of the reward could be self, other monkey, or neither (Chang et al., 2013a). (A) Left: recording sites on the medial wall, represented on a schematic of the different zones of the ACC. ACCg recordings were taken from a region that overlaps with both areas 24a/b and 32. Right: recording regions on a coronal plane of an MR image taken from Rushworth et al. (2004). (B) Example neurons from the ACCs (top) and ACCg (bottom). Shown are spiking activity profiles after monkeys made a reward allocation decision (aligned to Choice, left) and after the outcome was actually received (aligned to Reward, right). The ACCs neuron responded to “other” and “neither” after a choice and at the time the reward was delivered. By contrast, the ACCg neuron responded only to other reward and not to self or neither both at the time of choice and the receipt of the reward. (C) Proportion of neurons out of all neurons with a significant modulation to any decision or reward outcome that showed one of three potential reference frames. “Self- referenced” (red) neurons responded to rewarding outcomes referenced to self (whether self-received a reward or not) but did not distinguish between other or neither. These neurons could signal either received (predominantly in OFC) or foregone (predominantly in ACCs) reward for self. In blue are neurons that signaled reward for other only, and in purple are neurons that signaled reward for both self and other in a similar manner (but not to neither reward). The ACCg showed a significantly higher proportion of other-referenced and both-referenced neurons than either the ACCs or OFC, in which the majority of neurons were self-referenced.

Single-unit recording studies in macaques also suggest that the ACCg processes other-regarding information that is particularly important for social behavior, in a manner that is distinct from other regions of the cingulate cortex. Chang et al. (2013a) used a modified dictator game in monkeys, in which the actor monkeys made a decision to share or withhold reward from a conspecific or a decision to reward the conspecific or no one (Figure 2). Using this design they were able to disentangle whether neurons responded to rewarding outcomes being delivered to self, other, no one, or combinations of conditions. As well as ACCs, they recorded from neurons in a relatively anterior portion of ACCg, approximately at the border of areas 24a/b and 32 (Figures 2A and 2B). They found that a significantly higher proportion of neurons in the ACCg responded to rewarding outcomes delivered to another, with many responding exclusively when another monkey was receiving a reward. Specifically, they found that approximately 60% of neurons that responded to *any* choice, cue, or reward outcome (approximately 25% of total neurons recorded) in the ACCg (Figure 2C) signaled when another monkey was receiving a reward (whether exclusively for another monkey [other-referenced] or in a mirrored fashion when either self or other obtained a reward [both-referenced]). Nearly half of these reward-sensitive neurons in ACCg only responded to the other monkey’s reward. While 25% may seem a small proportion of neurons, it is comparable with the proportion of neurons in the premotor cortex exhibiting “mirror neuron”-like profiles and yet such PMv neurons putatively influence behavior (Kilner and Lemon, 2013) and impact on electromyographic activity at several synapses distance (Catmur et al., 2011). In contrast, approximately 70% of reward-modulated neurons in the ACCs and 80% in the OFC were self-referenced and did not respond to any information about other monkeys. Thus, the proportion of neurons in the ACCg sensitive to other’s reward outcome, including those that exclusively use an other-oriented frame of reference, is striking and distinctive. This finding would therefore suggest that, compared to the ACCs and other cortical regions, the ACCg may play a more significant role in processing “other-oriented” information.

**Figure 3:**
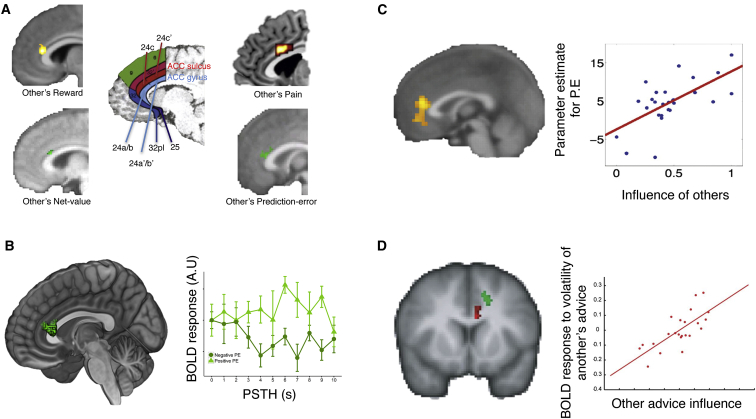
ACCg Responses to Other-Oriented Information in fMRI Studies (A) Results from fMRI studies in humans that have compared the processing of similar information for self and other. In these studies, activity was present for others’ high versus low reward probability (Lockwood et al., 2015; top left), the net-value (reward-effort) of another’s actions but not one’s own (Apps and Ramnani, 2014; bottom left), responses to others’ high pain more than low pain (from the meta-analysis of Lamm et al., 2011; top right), and others’ prediction error (Apps et al., 2015a). The central panel shows the locations of the ACC gyrus (blue) and ACC sulcus (red). Strikingly, these results all appear to fall in the same region the ACCg. (B) The response of the ACCg to others’ prediction errors when the subject monitored another’s actions. The graph shows the peristimulus time histogram plot of the BOLD response following trials in which the other person would have a positive PE (light green triangles) or a negative PE (dark green circles). (C) Response in the ACCg at the time of the outcome of decisions during a strategic social interaction task covarying with a PE updating beliefs about how the valuations of another will change (Zhu et al., 2012). The right panel shows that the extent to which this region signaled a PE (i.e., the parameter estimates for the covariation with the BOLD response) correlated with the degree to which subjects’ decisions were influenced by the other person. (D) Activity in the ACCg (red) to the outcomes of another’s advice or the outcomes of one’s own decisions (green) during a social interaction task in which a subject learned either from their own outcomes but also received advice from another. The right panel shows that the variability in the ACCg response at the time of the outcome of each action to how “volatile” the advice of another was, correlated with how influenced subjects’ behavior was by the advice of the other person.

Is there a similar specialization for other-regarding information in the ACCg in the human brain? Recently, a number of studies have asked whether ACCg processes self or other-oriented information. With a striking consistency, they have localized an ACCg region near area 24a/b that only responds to other-oriented information (Figure 3A). Crucially, all of these studies have highlighted that ACCg processes cues that are informative as to what events will impact another person even if it does not always process cues informative of events that will impact upon ourselves.

The ACCg is activated when processing cues that are predictive of others’ reward (Apps and Ramnani, 2014, Lockwood et al., 2015; Figures 2A and 2B) or provide information about whether rewarding outcomes are being delivered during social interactions (Apps et al., 2012, Apps et al., 2013a, Behrens et al., 2008, Zhu et al., 2012; Figures 3C and 3D) or when subjects process stimuli that are predictive of painful stimulation being delivered to another person (Lockwood et al., 2013, Lamm et al., 2011; Figure 2A). However, the ACCg is not activated by similar cues indicating that the subject themselves will receive painful stimulation or a reward. In addition, a study by Apps and colleagues (2013a) examined activity in the ACCg when processing the outcomes of either another’s decisions or the responses of a computer. Crucially, they found that the ACCg—albeit in a slightly more posterior region from the other neuroimaging and neurophysiological recording studies—responded to the outcomes of the other person’s decisions but not to the outcomes of a computer’s responses. Taken together, neurophysiological and neuroimaging evidence reveals that the ACCg responds to other-oriented information but not to information about ourselves or about non-biological agents.

#### The ACCg and the Motivational Value of Behavior for Another

As highlighted in the previous section, there is accumulating evidence that the ACCg processes other-oriented information. Although teasing apart signals that are related to decisions, predictions, or outcomes can be challenging (Cai and Padoa-Schioppa, 2012), in the this section we review evidence that suggests that the ACCg may play an important role in processing cues that allow for predictions about cost-benefit evaluations to be made about the value of a behavior for another.

In the study by Chang et al. (2013a), not only did the ACCg activity reflect reward delivered to others, but it also, at earlier time points in each trial, reflected the monkeys’ expectations about the reward about to be received by the other. Specifically, the normalized response profile of ACCg neurons reflected reward that the other monkey would receive prior to their delivery, but it did not change when rewards were to be delivered to no one or just to the self (Figure 2B). Therefore, ACCg activity reflects predictions about the motivated state of a conspecific prior to the actual reward delivery.

Neuroimaging studies have highlighted that human ACCg activity is found exclusively for cues that are predictive of reward for others. Lockwood et al. (2015) examined activity at the time of cues that was predictive of a high probability or a low probability of a reward for the subject or for another person. Crucially, they found that activity in the ACCg was sensitive only to the probability of another receiving a reward.

Some neural circuits are not just sensitive to primary reward but they are also sensitive to other factors that determine the net value of a course of action, such as costs entailed by the action made to obtain reward. This certainly is the case for cingulate regions concerned with reward-guided decision making even in the absence of social context (Rudebeck et al., 2006a, Kennerley et al., 2009, Kolling et al., 2016), but is it also true of the ACCg? Apps and Ramnani (2014) used fMRI to examine activity at the time of cues that indicated whether the subject or another person would have to exert effort to obtain a reward (Figure 3A). They found ACCg activity covaried with net value (reward – effort) at the time of the cues, but only on trials when the other person would have to exert the effort to obtain the reward. Such an activity pattern is analogous to that seen in the ACCs in non-social reward-guided decision making (Kolling et al., 2016). Other studies have also reported activity in the ACCg at times when predictions can be made about the value of a behavior for another (Boorman et al., 2013, Jones et al., 2011). Importantly, this includes studies in which subjects are evaluating reward that will be received by others that are delayed in terms of their receipt–another key factor that devalues rewards (Nicolle et al., 2012).

How does ACCg activity manifest when there is a possibility of a net negative state for another individual? If the ACCg is truly signaling value, then its activity should decline even further in such situation, but if instead the ACCg is signaling the net motivational impact of another person’s state, then its activity will increase as the net negative impact increases (cf. Roesch and Olson, 2007). ACCg activity conforms with the latter pattern; it encodes motivational salience; cues indicative of affective states closely linked to motivation, such as fearful faces or pictures of others in pain, also increase activity in the ACCg in the same manner as rewarding stimuli (Rotge et al., 2015, Lockwood et al., 2013, Molenberghs et al., 2012). But, as highlighted above, activity in this region shows an opposing profile in terms of being sensitive to the costs of others’ actions.

This profile suggests that at the time of cues that are predictive of behavior by another, activity increases in the ACCg when the benefit associated with the behavior is greater and actions will be more motivated (invigorated). The prospect of negative states as well as positive ones can lead to similar motivation or invigoration and it is with this motivational factor that ACCg activity covaries. That is, activity increases when another will avoid pain or obtain a reward. However, activity in this region also decreases with costs that decrease motivation, such as the effort costs or temporal delay before a beneficial outcome is received. This suggests that the ACCg is signaling the motivational value of acting for another.

#### The ACCg and the Outcomes of Others’ Behavior

While many studies suggest that the ACCg responds to stimuli that are predictive of events that impact another, the ACCg also appears to be sensitive to the *outcomes* of decisions that impact upon another. A large number of EEG and fMRI studies have shown during social interaction tasks that the ACCg signals when the outcomes of others’ actions are unexpected (Koban et al., 2010, Shane et al., 2008). As highlighted above, Chang et al. (2013a) identified that a group of reward-sensitive neurons in the macaque ACCg signal only when reward will be delivered to another and not to no one or ourselves. In humans, Behrens and colleagues (2008) asked participants to make decisions on the basis both of the history of reward that they had experienced with different choices, and also on the basis of advice that they were given. When the participants were given feedback at the end of the each trial, they were able to update both their estimates of action values and their estimates of how good the advisor was. The weight of influence, or impact, of the feedback information on these two updates was associated with activity change in the ACCs and ACCg, respectively; the activity increases were, respectively, proportional to the action learning rate parameter size and the advisor learning rate parameter size in the model used by Behrens and colleagues. This would point, therefore, to the ACCg playing an important role in other-oriented learning—learning about the behavior of others, and perhaps also, to learning about the value other’s place on behavior.

Other studies suggest that not only does the ACCg signal information at the time that outcomes are delivered to other people, but it signals when other people’s expectations about the consequences of their actions are revealed as erroneous. Such signals are typically referred to as prediction errors (PEs), a signal for tracking the statistical properties of the environment (Schultz, 2006). In the ACCg, the statistical properties of the contingencies between stimuli and rewarding outcomes for others are tracked by PEs. As reported above, Apps et al. (2013a) found that the ACCg was active when the outcomes of another’s actions are revealed to a subject. Specifically, they showed that ACCg activity signaled when the outcome of another’s action was discrepant from the predicted outcome. However, it signaled this PE only for the unexpected outcomes of another’s action and not for the outcomes of a computer. ACCg activity has also been found to covary with PEs when monitoring the outcomes of another’s strategy (Zhu et al., 2012) as well as when updating beliefs about other people’s strategies (Hampton et al., 2008). Crucially, all of these studies point to the ACCg being engaged when monitoring others’ outcomes and suggest that this region may signal “outcome”-related PEs (OPEs).

#### The ACCg and Monitoring Others’ Actions

Recent evidence suggests that the ACCg may also signal prediction errors (PEs) while we monitor the actions of another person, not only when viewing the outcomes of their actions. Apps et al. (2015a) asked subjects to act like a teacher and monitor the actions of another person (student) engaged in learning stimulus-response associations by trial and error (Figure 3B). As the subjects had learned all of these associations themselves, when they saw the action of the other person they could infer a PE for how different another’s prediction was from the actual outcome the subject knew they would receive. They found activity in the ACCg signaled this PE (Apps et al., 2015a). In other studies, the ACCg was found to respond more strongly when observing others’ actions that have unfamiliar or unusual kinematics (Casile et al., 2010, Maffei et al., 2015). It could be postulated that such a response in the ACCg might reflect the signaling of a discrepancy between the expected and actual kinematics of another’s action. That is, the ACCg may signal a PE when monitoring another’s movement, coding for the difference between an expectation about the value of a movement for another and the actual value of the movement executed.

#### Domain Generality in the ACCs

In contrast to the ACCg, the adjacent ACCs (or dACC as it is often called) does not seem to have such a specialization for other-oriented information. Based on single-unit recordings, lesion studies, and neuroimaging approaches (see Silvetti et al., 2014 or Kolling et al., 2016 for reviews), the ACCs, including its anterior perigenual portion, signals the value of our own behavior (Amemori and Graybiel, 2012), and updates those estimates when feedback suggests that they may have been incorrect (Procyk et al., 2016). As a result of these findings, many attempts have been made to develop domain-general models of the contributions of this region to cognitive processing (Alexander and Brown, 2011, Holroyd and McClure, 2015, Holroyd and Yeung, 2012, Silvetti et al., 2014, Verguts et al., 2015).

In studies directly investigating social information processing, there is mixed evidence as to how important a role the ACCs plays in processing social information. In human neuroimaging studies, the ACCs is activated when painful stimulation is delivered to either ourselves or other people. However, it also responds to the unexpected outcomes of both other people and computers (Apps et al., 2013a). This suggests that ACCs processes information regardless of whether it pertains to another biological agent or not. In Chang and colleagues (2013a), a study discussed in the preceding sections, the vast majority of ACCs neurons differentiated between reward delivered to self and other but the same neurons also similarly differentiated between conditions in which the self or no one obtained a reward. In fact, within the ACCs very few neurons distinguished between reward delivered to the other or to no one (Figure 2B). This would suggest that neurons in this region predominantly respond to “foregone” reward. That is, these neurons code for when a reward is not going to be delivered to ourselves. This would place these neurons within a “self” reference frame and not an “other” frame of reference.

In contrast, recent studies recording from the ACCs during more complex social interaction tasks could be interpreted as suggesting that neurons in this region signal other-oriented information. Yoshida et al. (2012) found neurons in the ACCs that responded when another monkey made an error during a reward incentivized action selection task. While this could be interpreted as reflecting “other-oriented” information, it is important to note that the subject’s own outcome and subsequent behavior was critically dependent on the action selection of the other monkey. A failure of the other monkey to act appropriately would therefore lead to an absence of reward for the monkey themselves. Thus, this finding is remarkably consistent with that of Chang et al. (2013a), who showed that the majority of responses in the ACCs reflect a “foregone” reward for ourselves in a self-referenced manner.

In another study, Haroush and Williams (2015) recorded from neurons in ACCs as monkeys played the prisoner’s dilemma game with another monkey. They found neurons that were predictive of the other monkeys’ choices of whether to cooperate in the iterative game. While these signals may be interpreted as reflecting the other-oriented process, it is also equally plausible that these signals may be self-referenced. With differential probabilities of the monkey themselves receiving a reward depending on the history of choices of the two monkeys, these neurons could be reflecting a prediction of a foregone reward as in Chang et al. (2013a). These neurons may therefore reflect the updating of the first monkey’s decision-making strategy on the basis of the other’s previous actions, which is critical for social interactions.

While this notion of domain generality might suggest that the ACCs has limited specialization for social information processing, it is important to consider the connections highlighted in previous sections between the ACCg and other areas of the brain. As noted, the ACCg has strong connections to the OFC, ventral striatum, and ACCs. Without doubt, these regions are important for guiding behavior, including social interactions (Azzi et al., 2012, Burke et al., 2010, Watson and Platt, 2012). However, neuroimaging and neurophysiological evidence suggests that they may not process information in the same reference frame as ACCg (Lee and Seo, 2016, Chang et al., 2013a, Azzi et al., 2012, Báez-Mendoza et al., 2013). Moreover, the ACCg is also connected to portions of the dmPFC and TPJ that have been shown to signal “other-oriented” information in a variety of different tasks (see Lee and Seo, 2016 for a review). This therefore places the ACCg within a distributed network that guides social behavior, with the ACCg processing information about the level of motivation of others agents during social interactions.

#### The ACCg and Variability in Social Behavior

Social abilities vary considerably even between healthy individuals (Bird and Viding, 2014). Notably, the structural and functional properties of the ACCg have been linked to variability in both social behavior and factors that influence socio-cognitive abilities.

**Figure 4:**
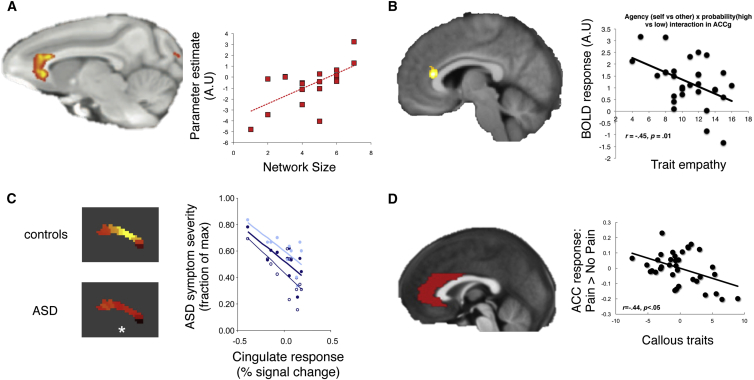
Variability and Pathology in ACCg Activity to Other-Oriented Information (A) Resting-state activity in the macaque ACCg (left) correlates with social network size (right; x axis: size of social group, y axis: strength of resting-state connectivity between ACCg and TPJ/STS) (Sallet et al., 2011). (B) The extent to which the ACCg (left) exclusively processes the expected value of others’ reward correlates with self-reported levels of emotion contagion (a component of empathy). The graph to the right shows the extent to which the ACCg signaled an interaction between probability of reward and agent identity (self versus other). In this graph, the interaction term (BOLD response) correlated with emotion contagion (trait empathy, x axis). Higher levels of empathy were related to greater specialization of the ACCg for processing others’ reward and not subjects’ own reward (Lockwood et al., 2015). (C) Differences in ACC activity between healthy controls and a high-functioning ASD group during a social interaction task (Chiu et al., 2008). Patterns of activity in controls and ASD (left) were different at a moment in time when subjects could predict the consequences of their actions on the reward that would be obtained by another. The response of the cingulate at that moment in time, correlated with ASD symptom severity (right). (D) Activity in the ACC to the pain of others (pictures of others’ in pain: y axis) is negatively correlated with callous (psychopathic/ICU-callous traits) traits (x axis) in children with conduct problems (Lockwood et al., 2013). Although responses in (C) and (D) were not localized specifically to the sulcus or gyrus, they do provide evidence that links together ACC and impairments in social behavior.

As highlighted above, the connectivity of the ACCg has been shown to reflect social group size (Sallet et al., 2011) (Figure 4A). There is also evidence that activity in the ACCg in response to other-oriented information—and particularly to stimuli that carry information about the motivational value of behavior for another—varies with trait levels of social abilities. Levels of our ability to empathize with others have been shown to be correlated with activity in the ACCg when processing information about pain being delivered to others (Lamm et al., 2011) or cues that are instructive of the probability of another receiving a reward (Lockwood et al., 2015) (Figure 4B).

There is also evidence that variability in the ACCg response is linked to social behavior during tasks in which people interact. Specifically, the extent to which activity in the ACCg responded to feedback about the advice of another is related to variability in the degree to which people are influenced by that advice (Behrens et al., 2008; Figure 3C). In addition, the extent to which ACCg signals information at the time of the outcomes of others’ actions, and particularly the degree to which this region signals PEs, correlates with how influenced by others’ behavior people are during competitive interactions (Zhu et al., 2012; Figure 3D).

These findings therefore suggest that while the response of the ACCg in the typical population is predominantly “other-oriented,” the degree to which the ACCg processes information in this reference frame varies between individuals. As a result, there is considerable variability in the response of the ACCg to predictions about the value of a behavior for another and also to the extent to which PEs update our predictions about other people. These studies point to individual differences in the response of the ACCg or the degree to which the response is in an other-oriented frame of reference (Chang et al., 2013b) being closely linked to variability in the ability to understand the value of a behavior for another individual.

#### Disorders of Social Cognition and the ACCg

In clinically diagnosed psychopathy and ASD, there is also evidence of disruptions to the structural and functional properties of the ACCg. Post-mortem evidence has highlighted changes in the histological properties of the ACCg and ACCs as one of the most replicable differences between the brains of individuals with ASD and healthy control individuals (Zikopoulos and Barbas, 2013). Resting connectivity of an area including the ACCg and also ACCs has also been shown to be different in a large sample of individuals with ASD compared to a matched control group (Balsters et al., 2016). It has also been shown that symptom severity in ASD is correlated with activity in the ACC during an economic exchange with another (Chiu et al., 2008; Figure 4C). In addition, psychopathic individuals and children who are rated as highly callous show differences in gray matter volume in the ACCg and connectivity between this region and other regions involved in processing social information such as the AI and the AON (Bird and Viding, 2014, Blair, 2013, Ly et al., 2012).

Existing evidence suggests that the functional properties disrupted in the ACCg in these disorders may be linked to the processing of information about the pain another is receiving. Activity in the ACCg to the pain of others has been shown to be attenuated in psychopathic individuals and those who exhibit callous traits (Anderson and Kiehl, 2012, Carré et al., 2013, Lockwood et al., 2013; Figure 4D). Similarly, the response of the ACCg to stimuli that indicate another is in pain is reduced in ASD compared to healthy controls (Fan et al., 2014). Prominent theories of ASD have also argued that deficits in social behavior may arise from the inability to effectively process motivation related information during social interactions (Chevallier et al., 2012).

There is therefore evidence to suggest that the ACCg may be a key region that is disrupted across different disorders of social cognition. Moreover, the deficit in the ACCg appears to be specifically tied to the processing of “other-oriented,” motivationally relevant information. Thus, while there have been limited specific investigations of the ACCg in disorders of social cognition, these results suggest that this region may be a fruitful target to examine in disorders of social cognition.

### Motivation and Vicarious Error Model: A Computational Framework of ACCg Contributions to Social Cognition

In the previous sections, we have highlighted how the ACCg may be the key region of the ACC that processes information in an “other-oriented” reference frame. Disruption to this region changes social behavior, a group of specialized neurons in this region respond to others’ reward, the region signals when information learned specifically about others is unexpected, and variability in this region is related to variability in social behavior. However, to date, there has been an absence of a theoretical framework that can inform future basic or clinical research. Moreover, while studies have hinted at the computational mechanisms that may operate within this region, this has never been formalized in a generalized manner that could characterize variability in behavioral or neural signals across a variety of tasks. This is in stark contrast to generalized models of dACC/ACCs, of which theoretical accounts are numerous and classical accounts suggest that the ACCs may be important for conflict monitoring, error detection, learning, and decision making (Alexander and Brown, 2011, Rushworth et al., 2012, Holroyd and Yeung, 2012, Shenhav et al., 2013, Ullsperger et al., 2014, Holroyd and McClure, 2015, Verguts et al., 2015, Kolling et al., 2016). Here, we suggest that the ACCg shares some of the computational properties of the ACCs but processes this information in an other-oriented frame of reference. In this next section, based on the extant literature and relevant research on the computations that underlie value-based motivation, we put forward a model that may be useful in explaining the previously identified contribution of the ACCg to processing information about others.

#### Understanding Others’ Motivation: A Key Social Process

In order to successfully interact with others, an agent must monitor the behavior of other individuals closely in order to predict the behaviors that they are likely to produce next and respond in the most adaptive manner (Adolphs, 2003, Brent et al., 2014, Fehr and Fischbacher, 2004, Frith and Frith, 2006). Crucially, to do so, an agent needs also to estimate the motivation of others. Accurately processing the level of motivation another has to obtain a desirable outcome (i.e., vicariously processing the value of a behavior for another individual) is a fundamental ability that is distinct from many other more frequently discussed components of social cognition (e.g., theory of mind, perspective taking, or empathizing) (Frith and Frith, 2006, Bird and Viding, 2014). For example, when I see someone running fast, this tells me that they are highly motivated at that moment in time. The vigor (speed) of their behavior therefore informs how motivated they are. However, it does not necessarily provide information on what their mental state is, nor is it necessarily informative as to their current mood. That is, we are able to infer whether someone is highly motivated or not, but their level of motivation can be orthogonal to the inferences we can make about their cognitive or affective state. Tracking another’s motivation may therefore be a key, but often neglected, component of social cognition.

The level of motivation or vigor in another’s behavior can therefore be thought of as a key piece of information that must be tracked during social interactions. Importantly, it can also be quantified. Anything that increases vigor can be thought of as motivating behavior toward a beneficial outcome that is being sought (e.g., obtaining a reward or avoiding pain), and anything that decreases vigor when there is a highly beneficial outcome offered can be thought of as a cost. This can be quantified as a “value” signal, with the benefits of moving fast traded off against the costs (Manohar et al., 2015). As argued in previous sections, we suggest that ACCg is sensitive to factors determining the motivation of others (their costs and benefits). There is little evidence that many other brain regions signal as much information about the reward or pain that is, or may be, received by another apart from perhaps the AI, a region to which the ACCg is strongly connected (Lamm et al., 2011, Mesulam and Mufson, 1982). This includes frontal lobe regions linked to reward-guided decision making, such as vmPFC and ACCs, and other brain regions such as TPJ and dmPFC linked to other aspects of social cognition and meta-representation (Chang et al., 2013a, Lee and Seo, 2016).

#### Vicarious Motivation: The Value of Behavior for Another

**Figure 5:**
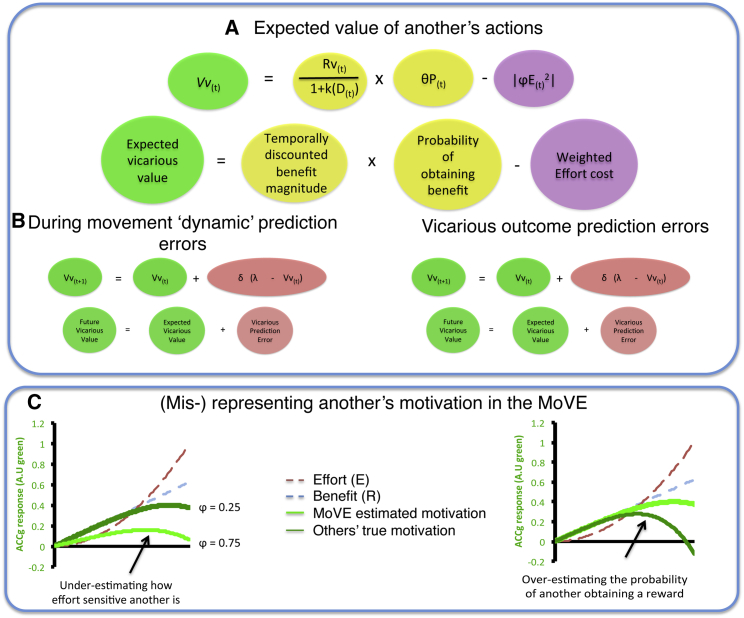
The MoVE Model (A) Prior to another’s movement, the value of a behavior for another individual can be estimated. From this it is possible to compute how motivated a specific individual will be following a specific cue associated with a particular behavior pattern. That is, an observer can weigh up the costs and benefits of a behavior for a specific other individual and vicariously estimate the expected “value” of a behavior for that individual (Vv). The benefit (Rv) for another reflects the motivational benefit of acting. Thus, value increases if the magnitude of a reward is higher, but also if magnitude of painful stimulation will be greater. Thus, the magnitude (salience) of the potential outcome, regardless of its valence, increases “value” in this model. The magnitude of the benefit is temporally discounted (kD), by the probability of the behavior succeeding (P) and weighed against the effort cost of exerting the action (ϕE). (B) These estimates are updated by two different categories of prediction error (PE). Vicarious outcome prediction errors (vOPEs) update expectations about the value of a behavior for another based on the presence or absence of a beneficial outcome (i.e., Rv, D, or P are updated); vicarious dynamic PEs (vDPEs), errors in the estimation of another’s motivation based on their movement kinematics and also update estimates of the motivation of another. These error signals can update the parameter estimates for another agent (i.e., K, θ, and ϕ), although we note that these idiosyncratic motivational parameters are not “stored” in the ACC, they influence estimates of how motivated a specific other individual may be. As a result, estimates of the motivation of another agent are updated by inferences about the internal states that guide another’s behavior. (C) There are multiple possible ways in which under- or over-estimation of parameters relating to the other individual can lead to an inaccurate estimate of how motivated the other individual will be, leading to inaccurate planning of one’s own behavioral response. Two examples are represented here graphically. First (left), we show that over-estimating how sensitive another will be (ϕ = 0.75) to the true effort (E) leads to a reduction in how motivated we infer another will be (light green) compared to their true level of motivation (dark green). Second (right), we show that under-estimating the probability (θP) that another will obtain a benefit leads to an under-estimation of their motivation (dark green) compared to their actual level of motivation (light green). Such changes illustrate how inaccurate parameter estimates can lead to misrepresentation of another’s motivation.

We break vicarious motivation (Figure 5) processing down into the three distinct phases. (1) We must infer and represent the expectations another has about the value of their own behavior in terms of the beneficial outcome they desire for themselves, and the costs they will incur to obtain the outcome. This vicarious value (Vv) can either be derived from exogenous cues from the environment that have been learned to be predictive of costs and benefits for others, from communication directly, or from cues such as facial expressions. (2) The Vv ascribed to others’ behaviors are updated dynamically, online *during the monitoring* of their actions; and (3) the value of cues associated with such behaviors are updated when a subsequent outcome reveals that the initial expectation at the time of a cue was erroneous (i.e., when we learn that the value of a particular cue for another is different from what we had expected). In this section, we outline how a model derived from computational accounts of value processing and motor control may be useful for quantifying the mechanisms underlying the vicarious processing of motivation. From henceforth, we refer to this computational framework as the Motivation and Vicarious Error model (MoVE).

As noted in previous sections, the ACCg appears to process information that increases the value of acting (e.g., avoiding pain or approaching reward) and also that which decreases value (e.g., effort or delays). Computationally, we therefore characterize a prediction of how motivated another is as processing the vicarious value (Vv) of an action they will perform. This Vv is dependent upon the magnitude of the benefit (R) for another individual. This benefit can be thought of as being always positively valenced or unsigned (i.e., regardless of the positive or negative valence of the outcome, the Vv increases with the magnitude of the outcome such as greater pain or reward). So avoiding a large loss, obtaining a large reward, or avoiding a significant amount of pain would all reflect a large benefit for another person and thus increase “value.” The benefit magnitude is devalued by three distinct costs: (1) the temporal delay (D) before another receives the benefit (Charnov, 1976, Mazur, 1985, Shadmehr et al., 2010, Schultz, 2006, Wolpert et al., 2003); a benefit received in a week is valued as worth less than the same benefit received immediately; (2) the probability (P) of the benefit being received (if a particular behavior has a low probability of resulting in the desired benefit, then motivation to execute that behavior will likely be low and so reaction times and movement velocities will be slower; Wolpert et al., 2003, Schultz, 2006, Manohar et al., 2015); and (3) the effort that must exerted in order to obtain the beneficial outcome (E). This “effort” is determined by the amount of control that must be exerted during the performance of the behavior (Wolpert et al., 2003). The greater the effort to be exerted, the more the behavior is devalued (Manohar et al., 2015, Verguts et al., 2015).

This model therefore predicts that activity in the ACCg will be highest for a highly beneficial behavior for another, which is immediate, has a high probability of being received, and for which minimal effort needs to be exerted. However, each of these is weighed by a parameter that dictates how sensitive we believe another is to these sources of information (k,θ,Φ). That is, how impulsive someone is (k), how sensitive they are to probability (θ), and how averse they are to effort (Φ) all dictate how motivated they will be. The response of the ACCg therefore depends on how these sources of information are weighed, as well as the real delays, probabilities, and effort that another must exert. Importantly, we do not suggest that these specific characteristics—that define other people’s traits—are necessarily “stored” in the ACCg, merely that when monitoring another’s behavior, they can influence the valuation another places on a behavior and how invigorated their actions will be. These parameters reflect how different individuals may therefore value benefits differently. Through its connections with other regions that are important for learning about others’ traits and states, the ACCg may therefore form part of a network that allows the Vv to be constantly updated based on how much another weighs benefits against costs.

As outlined in the previous paragraphs, a prediction of how motivated another is can therefore be characterized in the following equation:

$$\text{Vv}=\frac{\text{R}}{1+\text{kD}}\text{×}\theta \text{P}\text{-}\left|\varphi {\text{E}}^{2}\right|.$$

This equation therefore represents the key properties of the MoVE, evaluating the costs and benefits of a behavior for another. As noted in previous sections, activity in the ACCg has previously been shown to be modulated by all of these features and to signal the net value of others’ behaviors.

#### Two Different PEs for Updating Estimates of Others’ Motivation

As highlighted in previous sections, the ACCg signals PEs both when the outcomes of others’ actions are processed, but also when monitoring other’s actions. Similarly, two distinct updating signals are crucial for dynamically controlling one’s own behavior and learning from exogenous feedback (Figure 5B). At the time of an outcome (feedback) of a behavior, a comparison can be made between the actual value of an action and the expected value of the action. When these are discrepant, PE signals drive learning based on the magnitude of the error (Schultz, 2006, Sutton and Barto, 1998). These PEs are scaled by learning rate parameters that dictate how much is learned from a PE. The outcomes of others’ actions reveal whether expectations about the value of a behavior for that individual were accurate. At the time of the outcome of another’s behavior, we can compare our expectation about the value of an outcome of a particular behavior for another to the value of the actual outcome they receive. When there is a discrepancy, vicarious outcome PEs (vOPEs) update our expectations of the level of motivation of another in the future following similar cues in the environment. That is, if we observe another obtaining a reward after they have performed an action, having never previously been rewarded for that behavior, we will come to expect they will be more motivated to perform the action in the future.

Control theories also postulate another form of PE signal that occurs *during* movements. During our own actions, we monitor their consequences and correct errors in our movements by identifying discrepancies between expected and actual movement kinematics (Wolpert et al., 2003). During actions we can also dynamically update our evaluations of others’ motivations on the basis of the properties of the others’ movements that are witnessed, such as the speed (vigor) of their movement (Wolpert et al., 2003). When monitoring another’s behavior, we can therefore compare our expectation about the value of a behavior—and an expectation about the vigor of their movement—with the actual behavior we observe. These can therefore be thought of as “dynamic” PEs as they code for errors during movements. If the movement is faster than predicted, then the model increases the estimate of the value the other agent is placing on behavior (i.e., their motivation is higher than was expected), whereas the opposite is true when the movement is slower than predicted. Such discrepancies between the actual and expected speed of another’s movements are updated by a vicarious dynamic PE (vDPE) that improves estimates of how motivated another currently is while executing a behavior.

Crucially, vDPEs and vOPEs can be used to update estimates of the effort required, as well as the probability of receiving a reward (Verguts et al., 2015). In the MoVE model, these two forms of PE update estimates of the value of another’s behavior.

The responses in the ACCg to the value of behaviors for others and to PEs that were discussed in earlier sections approximate the signals predicted by the MoVE model (Lockwood et al., 2015, Apps and Ramnani, 2014, Zhu et al., 2012, Apps et al., 2012, Apps et al., 2013a, Apps et al., 2015a). This model therefore provides a new framework for understanding previously identified signals in the ACCg during social interaction tasks. Notably this model also has the potential to quantify individual differences in people’s processing of the motivation of other individuals. This model may therefore be able to quantify and provide a mechanistic understanding of failures to accurately represent the motivation of others (Figure 5C).

#### Distinguishing the MoVE Model from Others

Notably, the MoVE model shares commonalities with other models that have been proposed that can account for strategic learning and reasoning during social interactions including models of simulation (Suzuki et al., 2012), strategic inference (Camerer and Ho, 1998, Christopoulos and King-Casas, 2015, Hampton et al., 2008) or trust (Behrens et al., 2008). There are two key differences between these models and the MoVE. First, these models are predominantly aimed at computing abstract information about the strategy of others, and how we ourselves should adapt our strategy to reach goals during social interactions. In the MoVE model, the aim is to account for much lower-level information about how motivated other people are. Rather than attempting to explain strategic or theory of mind type inferences, this model makes predictions simply about the value of a behavior for another individual that allows predictions about the invigoration of another’s movement to be optimized. Second, in no previous model has the effort cost to be exerted by another been included within a model. This is pertinent given the significant influence that effort costs have on motivation (Kurzban et al., 2013, Manohar et al., 2015, Verguts et al., 2015, Apps et al., 2015b). The MoVE model therefore makes predictions about a component of social behavior that is not directly considered or parameterized in any of these models and as highlighted in other sections have been shown to be tracked in the ACCg.

We note, however, that Behrens and colleagues (2008) did report activity in the ACCg at the time of the outcomes of decisions that scaled with the volatility (or the current parametric value of a dynamically changing learning rate) of another’s advice. While the MoVE model does not directly predict the response in the ACCg witnessed in that study, it may have approximated some of what was observed, as in many circumstances the two models make similar predictions. Future studies should therefore use tasks where the properties of the two models lead them to make distinct predictions that can be tested against the response of the ACCg to determine whether volatility needs to be incorporated into the outcome response present in the ACCg.

### Future Directions

By proposing a new conceptual framework of the contribution of the ACCg to social cognition, there are a number of potential avenues of research. From an anatomical perspective, perhaps the most interesting question is what are the precise locations of ACCg zones processing other-oriented information. To date, recordings from the gyrus of the cingulate cortex in macaques have not extended posterior to the genu of the corpus callosum. In contrast, neuroimaging studies show activity across the ACCg to social information in both more posterior and anterior locations. Future research should therefore examine whether the other-oriented zone of the ACCg lies just in a small circumscribed region or whether a large portion is concerned with social information. Moreover, it has recently been shown that in the ACCs outcomes delivered to different parts of the body evoke activity in different zones (Procyk et al., 2016). It would be intriguing to examine whether a similarly topographic representation of others’ outcomes is also evident in ACCg. For example, is feedback for another’s arm movement, as opposed to feedback for facial movements, represented in a different portion of the cingulate gyrus.

To fully support the framework we have proposed, it would also be important to demonstrate that single neurons in the ACCg respond in a manner predicted by the MoVE model. For example, it will be important to examine whether neurons in the ACCg signal PEs relating to the unexpectedness of the speed of others’ movements and also to the unexpected outcomes of their actions.

An interesting question, which was beyond the scope of this Review, is whether different components of computing the value for others’ behaviors can be tied to distinct neuromodulators. Past studies suggest dissociable roles for oxytocin, dopamine, and serotonin in influencing social behavior (Chang et al., 2015, Crockett et al., 2015). However, future research should examine the specific influences of each of these on the processing of costs and benefits in the ACCg when they are vicariously processed.

Finally, we believe this model and framework may be more generally useful for understanding variability in social abilities such as empathy, cooperation, competition, as well as disorders of social cognition. Do all disorders of social cognition have deficits in processing others’ motivation and, if they do, can they be parameterized by different components of the MoVE model? Future work using tasks tailored to understanding how motivation is tracked during social interactions may be able to tease apart distinct deficits in different disorders in which there is ACCg dysfunction.

### Conclusions

In summary, research across different species in terms of anatomical connectivity, neuroimaging, and neurophysiology support the notion that a region in ACCg—adjacent and dorsal to the genu of the corpus callosum in humans—plays a crucial role in evaluating the behaviors of others and in estimating others’ level of motivation. We review the anatomical and functional role of the ACCg in social processing and put forward a mechanistic, computational account of the contributions of the ACCg to social cognition. Our account predicts many of the responses observed in the ACCg including those recorded from individual neurons and in neuroimaging studies. In addition, the MoVE model is easily generalizable to other domains of complex social behavior in which it is also necessary to understand others’ motivation. Looking forward, this framework may help to characterize and provide a mechanistic understanding of disorders of social cognition, by parameterizing potentially dysfunctional components of social behavior.

## Author Contributions

M.A.J.A., M.F.S.R., and S.W.C.C. wrote the paper.
